# Involvement of miR-451 in resistance to paclitaxel by regulating YWHAZ in breast cancer

**DOI:** 10.1038/cddis.2017.460

**Published:** 2017-10-05

**Authors:** Wenrui Wang, Lingyu Zhang, Yangyang Wang, Yongxing Ding, Tiantian Chen, Yueyue Wang, Haifeng Wang, Yu Li, Kecai Duan, Sulian Chen, Qingling Yang, Changjie Chen

**Affiliations:** 1 Department of Biotechnology, Bengbu Medical College, Anhui 233030, China; 2 Clinical Testing and Diagnose Experimental Center, Bengbu Medical College, Anhui 233030, China; 3 Clinical Laboratory, The First People’s Hospital of Changzhou, Jiangsu 213000, China; 4 Department of Oncology, Bengbu Central Hospital, Bengbu 233030, Anhui China; 5 Department of Biochemistry and Molecular Biology, Bengbu Medical College, Anhui 233030, China

## Abstract

MicroRNAs (miRNAs) have been identified as major post-transcriptional regulators of the initiation and progression of human cancers, including breast cancer. However, the detail role of miR-451 has not been fully elucidated in breast cancer. In this study, we aimed to investigate the biological role and molecular mechanisms of miR-451 in drug resistance in breast cancer cell lines and in xenograft model. We show that miR-451 is decreased in human breast cancer specimens and in paclitaxel-resistant (PR) cells. Ectopic expression of miR-451 could inhibit the cell migration and invasion, promoted apoptosis, induced cell-cycle arrest Furthermore, tyrosine3-monooxygenase/tryptophan5-monooxygenase activation protein zeta (YWHAZ) was identified as a direct target of miR-451. Remarkably, the expression of YWHAZ is inversely correlated with the level of miR-451 in human breast cancer samples. Co-treatment with miR-451 mimics and YWHAZ-siRNA significantly enhanced YWHAZ knockdown in both SKBR3/PR and MCF-7/PR cells Moreover, miR-451 markedly inhibited expression of *β*-catenin via YWHAZ and subsequently inhibited downstream gene cyclin D1, c-Myc expression. The results of xenograft model *in vivo* showed that intratumor injection of miR-451 agomir induced a tumor-suppressive effect in SKBR3/PR drug-resistant xenograft model. Taken together, our findings suggested that miR-451 might be considered as important and potential target in paclitaxel-resistant breast cancer treatment.

Breast cancer (BC) is one of the most common malignant cancers in women worldwide.^1^ According to the American Cancer Society, an estimated 252 710 new cases of invasive BC will be diagnosed and 40 610 cancer deaths will be occurred in BC in 2017.^2^ The main carcinogenic factors, including genetic mutations, endocrine disorders, and decline in immune function may increase the risk of developing BC.^3^ Chemotherapy is one of the current main anticancer therapies for the treatment of BC which has shown to inhibit tumor growth and prolong patient survival.^4^ Paclitaxel has been recognized as the first-line therapy in BC treatment. However, its efficacy is often limited by the development of drug resistance. Hence, paclitaxel resistance (PR) is the main obstacle in the treatment of BC.^5^ It is necessary to explore the molecular mechanism of chemoresistance and subsequently find a novel strategy to overcome such limitations for achieving better therapeutic effects in BC patients.

MicroRNAs (miRNAs) are a group of short, non-coding, single-stranded small RNAs containing about 22–29 nucleotides.^6^ It is well known that miRNAs exert their regulatory functions by binding to the 3′-untranslated region (3′-UTR) of target mRNAs, leading to the degradation of the mRNA or translational inhibition of functional proteins.^7^ Interestingly, increasing evidence has demonstrated that miRNAs play important roles in the regulation of cell proliferation,^8^ apoptosis,^9^ EMT^10^ and metastasis^11^ in various human cancers including BC. In recent years, some miRNAs have been reported to be involved in the modulation of drug resistance-related pathways.^12, 13^ For instance, miR-21 was able to affect the response to both trastuzumab and chemotherapy, triggering an IL-6/STAT3/NF-*κ*B-mediated signaling loop and activating the PI3K pathway.^14^ Zhu *et al.* reported that downregulation of miR-27b-3p enhanced tamoxifen resistance in BC by increasing NR5A2 and CREB1 expression.^15^ In our previous work, we have revealed that upregulation of miR-125b or targeting Sema4C could serve as novel approaches to reverse chemotherapy resistance in BC.^16^

It has been reported that miR-451 was directly involved in the development of drug resistance of several types of cancers, such as non-small-cell lung cancer (NSCLC), colon carcinoma and glioblastoma.^17, 18, 19^ Although these studies have showed that the potential role of miR-451 in chemoresistance in human cancer, the function of miR-451 in BC remains poorly understood.

In this study, we explored the role of miR-451 *in vitro* and *in vivo*. The expression of miR-451 was significantly downregulated in BC samples compared to matched adjacent normal breast tissue. Furthermore, we found that the overexpression of miR-451 could inhibit the cell migration and invasion and promoted apoptosis *in vitro.* More importantly, ectopic expression of miR-451 or depletion of its target YWHAZ could enhance the sensitivity of PR cells to paclitaxel. Finally, we constructed SKBR3 xenograft model and SKBR3/PR drug-resistant xenograft model *in vivo*. Results showed that intratumor injection of miR-451 agomir induced a tumor-suppressive effect in SKBR3/PR mice. In summary, this study demonstrated that miR-451 expression was downregulated in BC via directly targeting YWHAZ. Our findings provided further evidence that miR-451 might be considered as an important and potential target in paclitaxel-resistant BC treatment.

## Results

### MiR-451 expression is significantly downregulated in breast cancer

**Figure 1:**
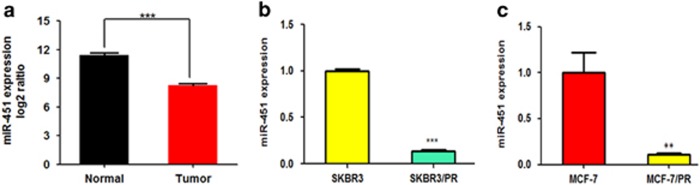
MiR-451 is downregulated in human BC samples and in PR breast cancer cells. (**a**) Expression of miR-451 in tumor tissues and matched adjacent normal tissues was revealed by miRNA-seq data provided by analyzing the Cancer Genome Atlas (TCGA) breast cancer miRNA sequencing data sets (normal, *n*=104; tumor, *n*=104). (**b** and **c**) Real-time RT-PCR assay was performed to detect expression of miR-451 in parental and PR cells. Data are shown as mean±S.D. (*n*=3). The symbols *, ** and *** represent great significant difference (*P*<0.05, *P*<0.01 and *P*<0.001) by two-tailed Student’s *t*-test

To identify the role of miR-451 in BC development, firstly, 104 samples of patients with BC that provided by TCGA data portal were detected in this study, as shown in Figure 1a, the expression of miR-451 was significantly downregulated in BC samples compared to matched adjacent normal breast tissue. Furthermore, to determine the identity of miRNAs that might contribute to the drug-resistant phenotype, a miRNA array was employed to characterize the miRNA signature of MCF-7 and drug-resistant MCF-7/PR cells. The differentially expressed miRNAs with two-fold changes in expression are shown in Supplementary Figure S1. MiR-451 showed the larger degree of downregulation (5.6-fold).To determine whether miR-451 might be involved in drug resistance in BC cells, we compared miR-451 expression between the paclitaxel-resistant cells and their parental cells. We showed that expression of miR-451 was significantly lower in paclitaxel-resistant cells than their parental cells. These results indicated that reduced miR-451 expression was a frequent event in human BC tissues and cells, which may be involved in breast carcinoma progression (Figures 1b and c).

### MiR-451 suppresses cell migration, invasion and induces cell-cycle arrest and apoptosis in breast cancer

**Figure 2:**
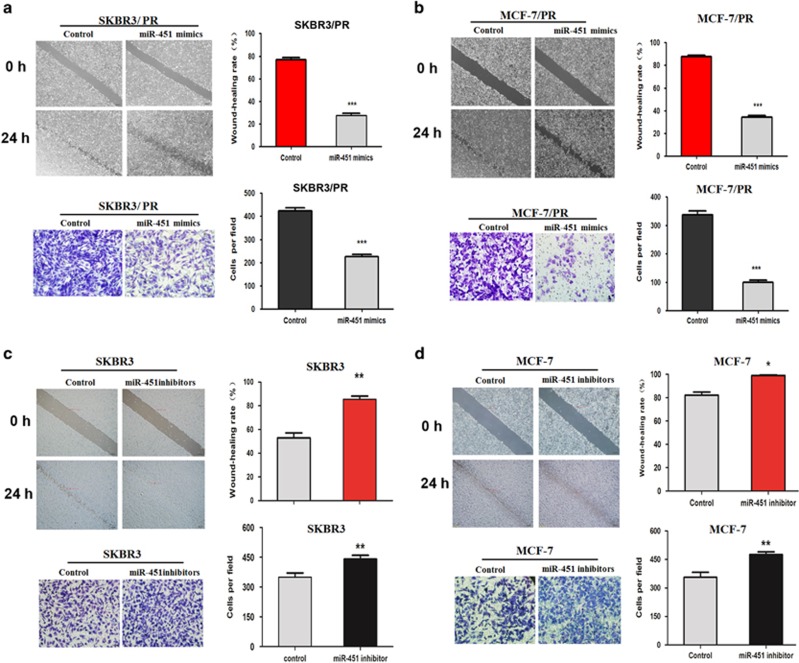
Effect of miR-451 on cell migratory, invasive activity *in vitro*. (**a** and **b**) Wound healing assay were performed to compare the migratory potential of SKBR3/PR and MCF-7/PR cells after transfected with miR-451 mimic. Representative image depicting the beginning (*t*=0 h) and the end (*t*=24 h) of the recording are shown. Invasion assay was conducted to measure the invasive capacity in SKBR3/PR and MCF-7/PR cells after transfection with miR-451 mimic. (**c** and **d**) Wound healing assay was performed to compare the migratory potential of SKBR3 and MCF-7 cells after transfected with miR-451 inhibitor. Representative image depicting the beginning (*t*=0 h) and the end (*t*=24 h) of the recording are shown. Invasion assay was conducted to measure the invasive capacity in SKBR3 and MCF-7 cells transfected with miR-451 inhibitor. Bars represent mean±S.D. and asterisks denote a significant difference (*P<0.05; **P<0.01 and ***P<0.001). Data are representative of at least three independent experiments

To investigate the biological role of miR-451 in BC progression, we performed *in vitro* gain and loss-of-function analyses in PR and their parent cell lines transfected with miR-451 mimics and inhibitor. The wound healing assay and transwell assay were conducted to investigate the cell migration and invasion, respectively. As shown in Figures 2a and b, miR-451 mimics significantly inhibited the cell invasion and migration in both SKBR3/PR and MCF-7/PR cells. By contrast, miR-451 inhibitor treatment led to increased cell invasion and migration in SKBR3 and MCF-7 cells (Figures 2c and d). In summary, these data suggested that miR-451 might play a critical role in cell migration and invasion in BC progression.

**Figure 3:**
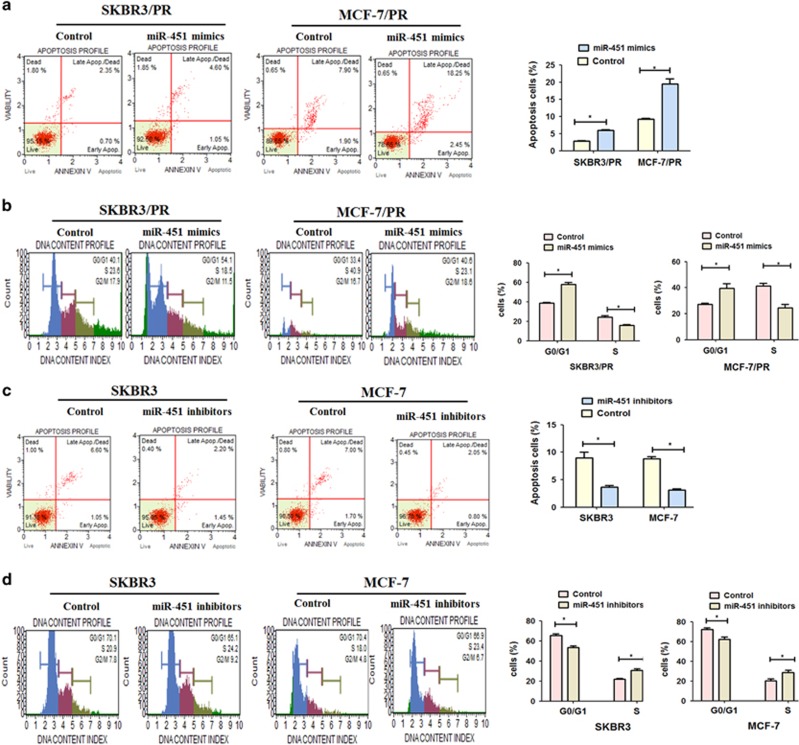
Effect of miR-451 on cell apoptosis and cell-cycle arrest *in vitro*. (**a** and **b**) Evaluation of the effect of miR-451 mimics on cell apoptosis and cell cycle in SKBR3/PR and MCF-7/ PR using Muse Cell Analyzer. (**c** and **d**) Evaluation of the effect of miR-451 inhibitors on cell apoptosis and cycle in SKBR3 and MCF-7 using Muse Cell Analyzer. Bars represent mean±S.D. and asterisks denote a significant difference (*P<0.05; **P<0.01 and ***P<0.001). Data are representative of at least three independent experiments

We subsequently evaluated the potential role of miR-451 in cell apoptosis and cell-cycle regulation by transfecting miR-451 mimics and inhibitor, respectively. Both SKBR3/PR and MCF-7/PR cells were transfected with miR-451 mimics, and the cell apoptosis was analyzed using flow cytometry. As shown in Figure 3a, the proportion of apoptotic cells transfected with miR-451 mimics was increased in SKBR3/PR cells compared with the control. Similar results were observed in MCF-7/PR cells. On the contrary, the proportion of apoptotic cells transfected with miR-451 inhibitors was decreased in SKBR3 and MCF-7 cells compared with the control (Figure 3c).

In addition, the alteration of the cell-cycle distribution following miR-451 overexpression in the two PR cell lines was also analyzed. As shown in Figure 3b, SKBR3/PR and MCF-7/PR cells transfected with miR-451 mimics showed a reduction of cells in S phase (from 23.6 to 18.5% for SKBR3/PR, from 40.9 to 23.1% for MCF-7/PR), whereas the percentage of cells in the G0/G1 phases increased (from 40.1 to 54.1% for SKBR3/PR, from 33.4 to 40.6% for MCF-7/PR).

### Prediction of YWHAZ as a target of miR-451

**Figure 4:**
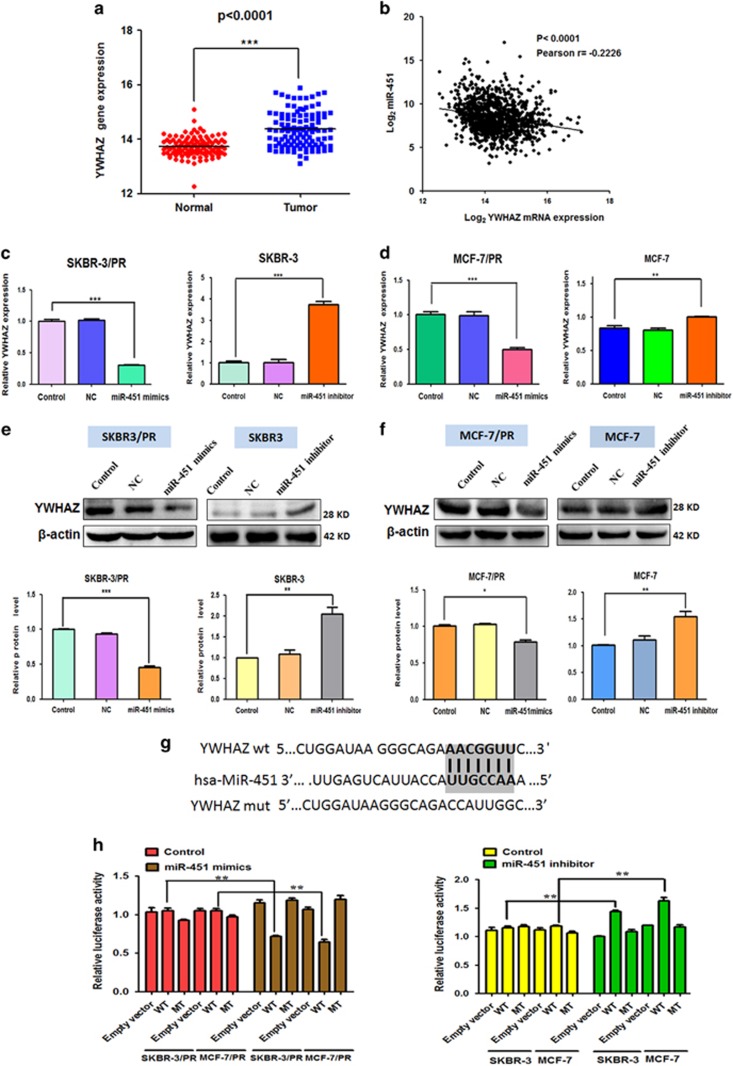
YWHAZ is associated with miR-451 expression. (**a**) mRNA expression of YWHAZ in tumor tissues and adjacent normal tissues of BC patients was revealed by mRNA-seq provided by TCGA. (**b**) Scatterplot depicts a significant inverse correlation between miR-451 and YWHAZ mRNA expression. (**c** and **d**) Real-time RT-PCR assay and (**e** and **f**) western blotting analysis were performed to detect the expression of YWHAZ at the mRNA and protein levels, in PR cells treated with miR-451 mimics and parent cells treated with miR-451 inhibitor,respectively. (**g**) Sequences of wild-type and. mutant target sites for miR-451 in YWHAZ are shown. (**h**) Luciferase reporter assays were performed to identify the binding of miR-451 to YWHAZ 3′-UTR in PR cells and their parent cells. MT, mutation; WT, wild type. Bars represent mean±S.D. and asterisks denote a significant difference (*P<0.05; **P<0.01 and ***P<0.001). Student’s *t*-test. Data is representative of at least three independent experiments

It has been well known that miRNA exerts its function via binding to the 3′-UTR of target genes through partial sequence homology.^20^ Therefore, to explore the molecular mechanism by which miR-451 modulates PR, publicly available algorithms (TargetScan, PicTar and miRanda) were used in combination to predict for potential targets of miR-451. Among these genes, YWHAZ was identified as a potential target of miR-451 and was selected for further experimental verification. The predicted interaction between miR-451 and the target site in the YWHAZ 3′-UTR is illustrated in Figure 4g. A highly conserved sequence was displayed that was complementary to the ‘seed sequence’ of miR-451 and was identified within the YWHAZ 3′-UTR.

### Validation of YWHAZ as a direct and specific target of miR-451

We analyzed the mRNA expression of YWHAZ in RNA-seq data of BC patients that were provided by TCGA data portal. Interestingly, we found that YWHAZ were upregulated in cancer tissues compared to adjacent normal tissues (Figure 4a). In addition, expression of YWHAZ mRNA and miR-451 exhibited a significant inverse correlation as calculated by Pearson correlation in BC patients (*r*=−0.2226, *P*<0.0001) (Figure 4b). These results supported that miR-451 targets to YWHAZ.

We consequently conducted further analysis to explore whether miR-451 regulated YWHAZ expression in PR cells. Transfected miR-45 mimics resulted in significant reduction of YWHAZ mRNA and protein, whereas miR-451 inhibitor treatment caused the upregulation of YWHAZ in SKBR3 and MCF-7 cells (Figures 4c–f). Furthermore, to validate whether YWHAZ is a direct and specific target of miR-451, miR-451 mimics and YWAHZ 3′-UTR wild type or 3′-UTR mutated luciferase reporter were transfected into SKBR3/PR and MCF-7/PR cells. Noticeably, the luciferase activity was significantly suppressed following co-transfection of miR-451 with wt-YWHAZ-3′UTR vector, but not in mutant YWHAZ-3′UTR in SKBR3/PR and MCF-7/PR cells (Figure 4h). Moreover, we observed that miR-451 inhibitor increased YWAHZ 3′-UTR wild-type, but not YWAHZ mutation, in SKBR3 cells and MCF-7 cells. These results indicated that YWHAZ gene was a direct target of miR-451 and might contribute to miR-451-mediated PR in BC.

### MiR-451 decreases the mRNA and protein expression level of *β*-catenin and relative genes of *β*-catenin signaling pathway *in vitro*

**Figure 5:**
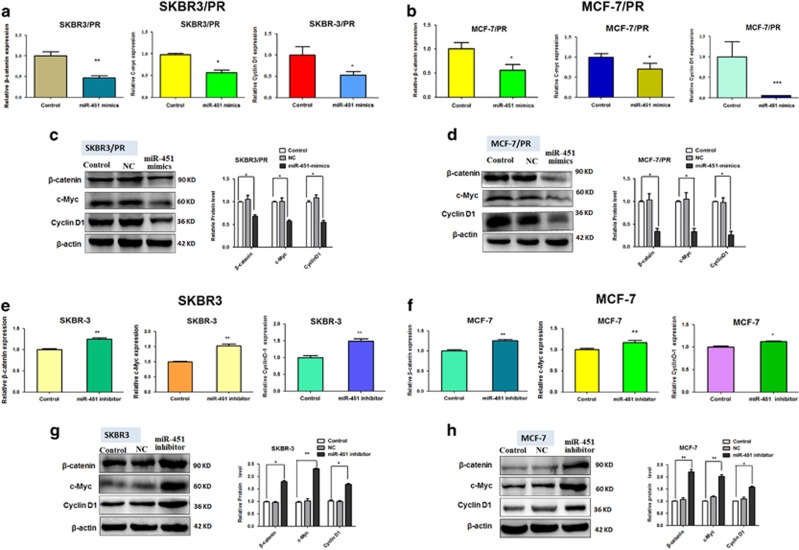
Effect of miR-451 on the mRNA and expression level of *β*-catenin and downstream targets c-Myc, cyclin D1. (**a** and **b**) Real-time RT-PCR analysis and (**c** and **d**) western blotting analysis was performed to detect the mRNA levels and expression level of *β*-catenin, c-Myc and cyclinD1 in SKBR3/PR and MCF-7/PR cells after miR-451 mimics treatment. (**e** and **f**) Real-time RT-PCR analysis and (**g** and **h**) western blotting analysis was performed to detect the mRNA levels and expression level of *β*-catenin, c-Myc and cyclinD1 in SKBR3 and MCF-7 cells after miR-451 inhibitors treatment. Bars represent mean±S.D.and asterisks denote a significant difference (*P<0.05; **P<0.01 and ***P<0.001). Data are representative of at least three independent experiments

Previous studies had shown that YWHAZ/*β*-catenin complex involved in drug resistance in cancer metastasis.^21^ In order to confirm miR-451 regulation of *β*-catenin expression via YWHAZ, the mRNA and protein levels of *β*-catenin and its downstream target genes were examined by qRT-PCR and western blot. As shown in Figures 5a–d, overexpression of miR-451 reduced *β*-catenin, c-Myc and cyclinD1 expression in both two PR cell lines, on the other hand, as shown in Figures 5e–h, downregulate miR-451 had the opposite effect and the expression of *β*-catenin, c-Myc and cyclinD1 increased in their parent cell lines.

### YWHAZ inhibition is required for miR-451-mediated effects

**Figure 6:**
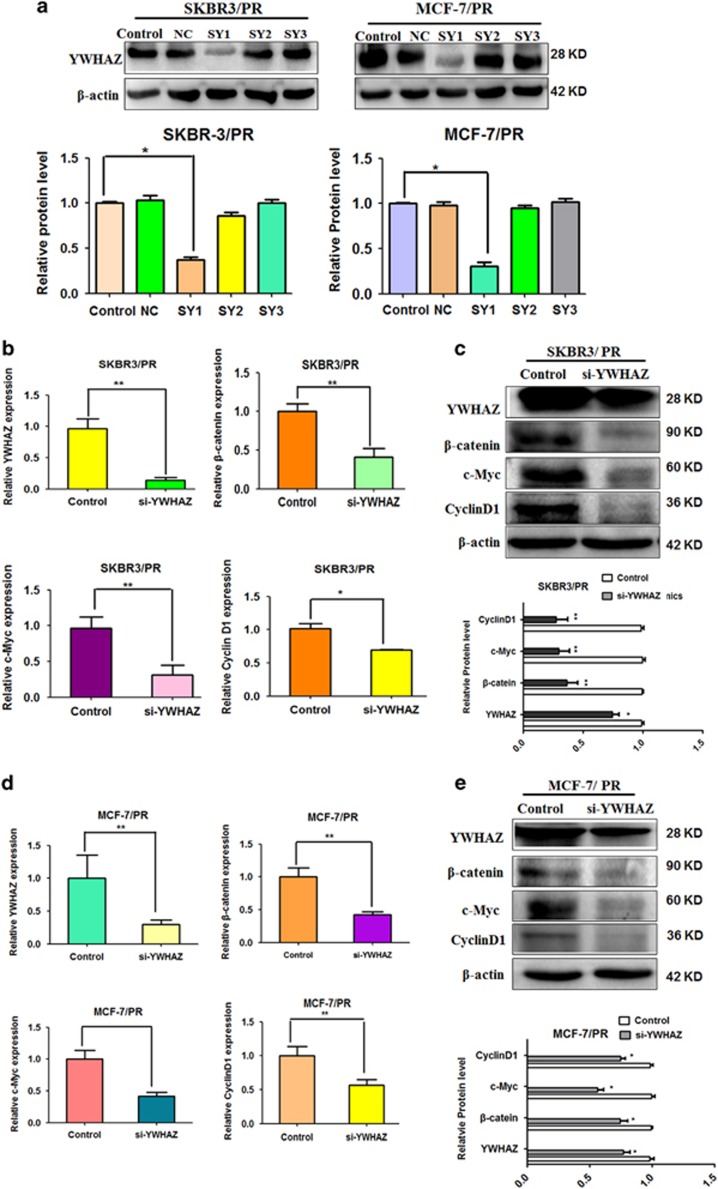
Downregulation of YWHAZ decreases the mRNA and expression level of *β*-catenin, cyclinD1 and c-Myc in both PR breast cancer cells. SY:siRNA-YWHAZ (**a**) Western blotting analysis was performed to measure the expression of YWHAZ in SKBR3/PR and MCF-7/PR cells after YWHAZ siRNA transfection. (**b** and **c**) SKBR3/PR cells transfected with control siRNA or YWHAZ siRNA were used for assessing the expression of *β*-catenin, c-Myc and cyclin D1 using Real-time RT-PCR(left panel) and Western blotting analysis(Right panel). (**d** and **e**) MCF-7/PR cells transfected with control siRNA or YWHAZ siRNA were used for assessing the expression of *β*-catenin, c-Myc and cyclin D1 using real-time RT-PCR (left panel) and western blotting analysis (right panel). Bars represent mean±S.D. and asterisks denote a significant difference (*P<0.05; **P<0.01 and ***P<0.001). Data are representative of at least three independent experiments

To determine whether YWHAZ plays a key role in drug resistance in BC cells, we depleted the YWHAZ using its specific siRNAs in SKBR3/PR and MCF-7/PR cells. As shown in Figure 6a, we found that YWHAZ siRNA1 transfection markedly inhibited YWHAZ expression. Thus, we used YWHAZ siRNA1 to conduct the following experiments. YWHAZ mRNA level was also significantly downregulated by YWHAZ siRNA treatment in both PR cells (Figures 6b and d). A similar decrease was observed when YWHAZ protein expression was determined by western blot (Figures 6c and e). Furthermore, we measured the expression level of *β*-catenin, cyclinD1 and c-Myc. Our RT-PCR results also showed that depletion of YWHAZ decreased the mRNA level of *β*-catenin, cyclinD1 and c-Myc in both PR cells (Figures 6b and d). With respect to protein expression, depletion of YWHAZ decreased the level of *β*-catenin, cyclinD1 and c-Myc in both PR cells (Figures 6c and e).

**Figure 7:**
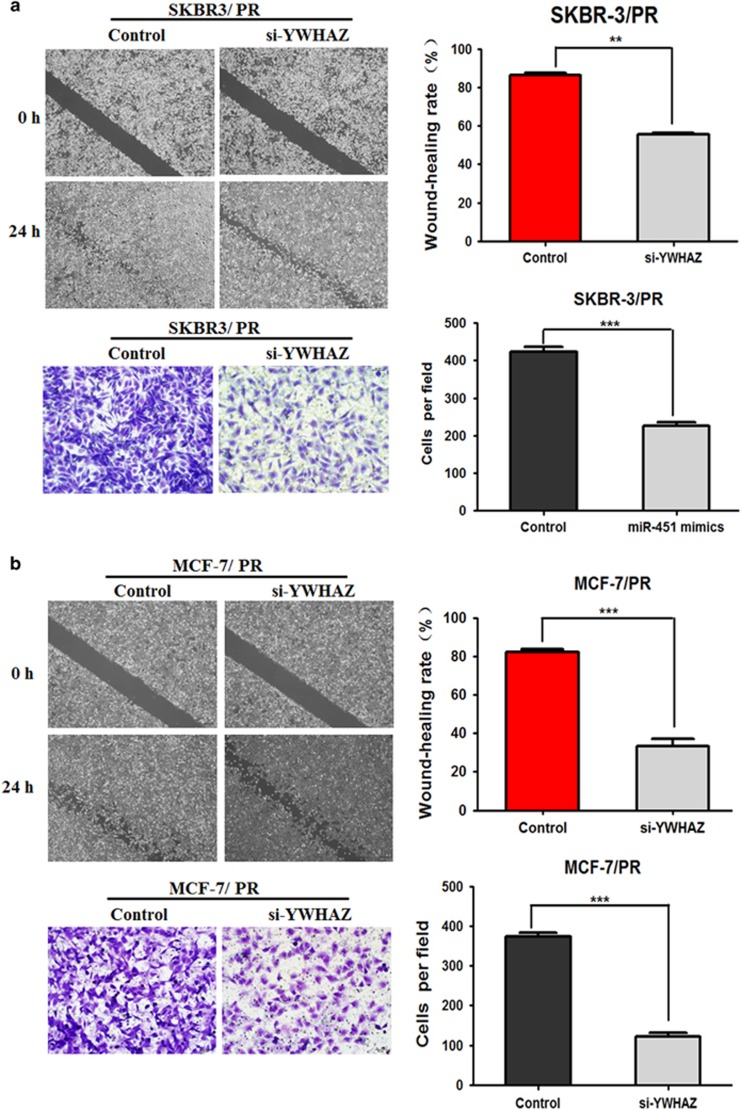
Downregulation of YWHAZ inhibited cell migration, invasion in both PR BC cells. (**a**) Transfection of SKBR3/PR with YWHAZ siRNA inhibited cell migration and invasion. (**b**) Transfection of MCF-7/PR cells with YWHAZ siRNA inhibited cell migration and invasion. The results were showed as mean±S.D. of three independent experiments

To clarify the importance of the role of YWHAZ for miR-451-mediated inhibition of cell migration and invasion, cells were transfected with YWHAZ siRNA. We found that YWHAZ siRNA transfection led to inhibition of cell migration and invasion in both SKBR3/PR and MCF-7/PR cells (Figures 7a and b). In summary, these results suggested that YWHAZ downregulation might be one important cause for the decrease in cell migration and invasion.

**Figure 8:**
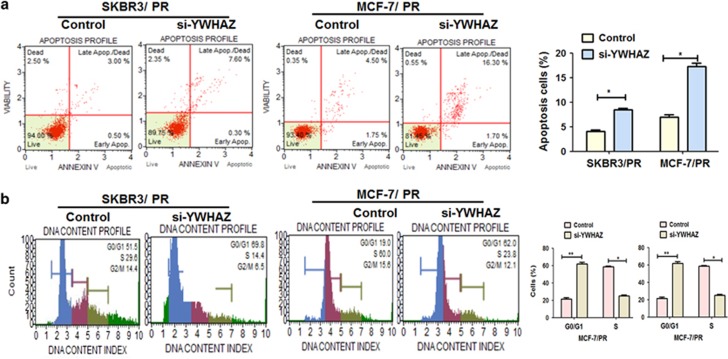
Downregulation of YWHAZ induced cell apoptosis and cell cycle in both PR BC cells. (**a**) Evaluation of the effect of YWHAZ-siRNA cell apoptosis in SKBR3/PR and MCF-7/PR using Muse Cell Analyzer. (**b**) Evaluation of the effect of YWHAZ-siRNA on cell cycle in SKBR3/PR and MCF-7/PR using Muse Cell Analyzer. Data are shown as mean±S.D. (*n*=3). The results were showed as mean±S.D. of three independent experiments

Next, we analyzed cell apoptosis using flow cytometry. As shown in Figure 8a, PR cells transfected with YWHAZ siRNA had a significantly higher apoptosis rate than the NC group. Furthermore, we examined the effect of YWHAZ siRNA on cell-cycle profiles. The numbers of cells accumulated in the G0/G1 phase were increased while those in S and G2/M phases were decreased when transfecting with YWHAZ siRNA (Figure 8b).

### Synergistic effect of combined miR-451 and YWHAZ-siRNA

Combining miRNA with siRNA is an attractive approach that may allow a ‘boosting’ effect for targeting oncogenic pathways.^22^ In this study, we investigated whether dual inhibition of YWHAZ by si-YWHAZ and overexpression of miR-451 showed synergistic effect.

**Figure 9:**
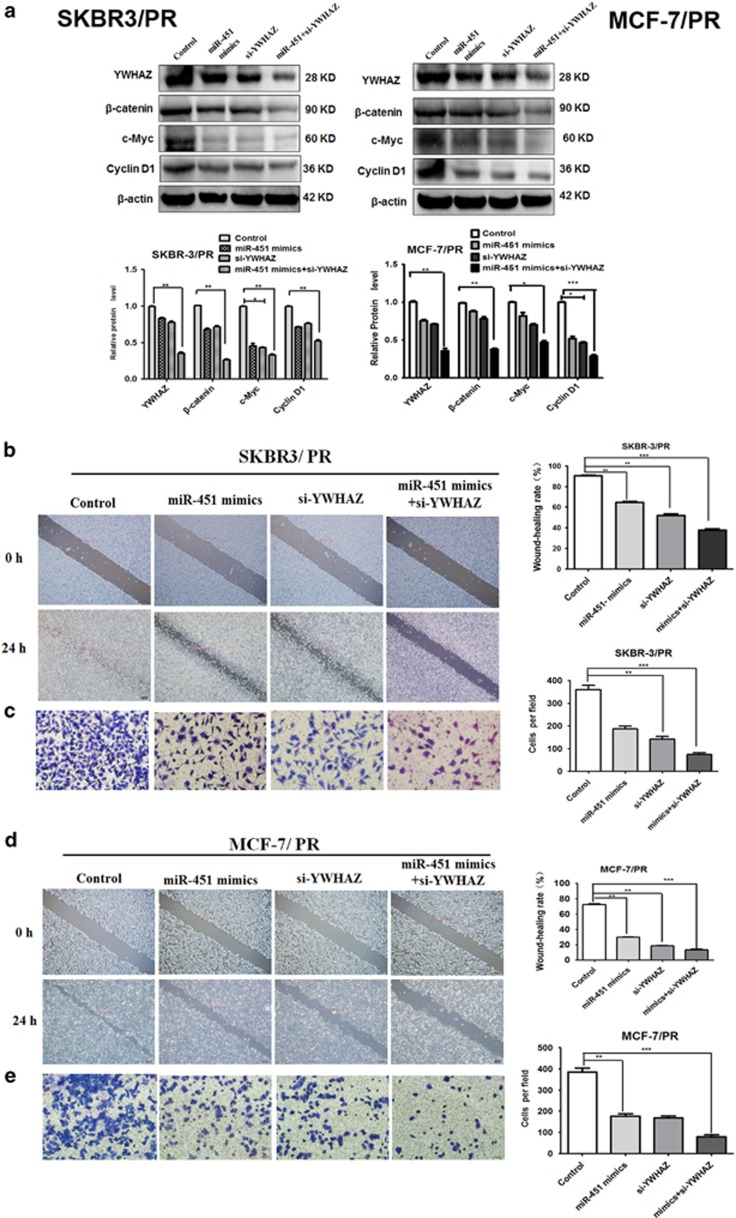
Combination of miR-451 and siRNA-YWHAZ treatment showed enhanced YWHAZ inhibition *in vitro*. (**a**) Western blotting analysis was performed to measure the expression of YWHAZ in SKBR3/PR and MCF-7/PR cells after treatment with YWHAZ siRNA or a combination of both. (**b** and **c**) Representative images showing effect of different combination treatments on SKBR3/PR and (**d** and **e**) MCF-7/PR migration and invasion. Cells were counted in five random fields per well after 24 h for migration and invasion and the percent migratory or percent invasive cells were calculated. The results were showed as mean±S.D. of three independent experiments

As shown in Figure 9a, co-treatment with miR-451 mimics and YWHAZ-siRNA significantly enhanced YWHAZ knockdown in both SKBR3/PR and MCF-7/PR cells. To further refine these correlations and to determine the functional consequences of this combination, we performed *in vitro* cell migration and invasion analysis after miR-451 mimics/si-YWHAZ treatment in both PR cells. Treatment with individual therapy significantly decreased the migratory and invasive capabilities of both PR cell lines in comparison to control treatment (Figures 9b–e). However, combination of both treatments further enhanced the inhibition of cell migration and invasion (Figures 9b–e). The combination therapy showed synergistic inhibition of cell invasion (ratio of expected: observed Fa is 1.2 for SKBR3/PR and 1.4 for MCF-7/PR cells respectively), but not in migration. These results together suggested the functional significance of miR-451 and support enhanced efficiency of miR-451/si-YWHAZ combination *in vitro.*

### Role of miR-451 in modulating paclitaxel resistance *in vivo*

**Figure 10:**
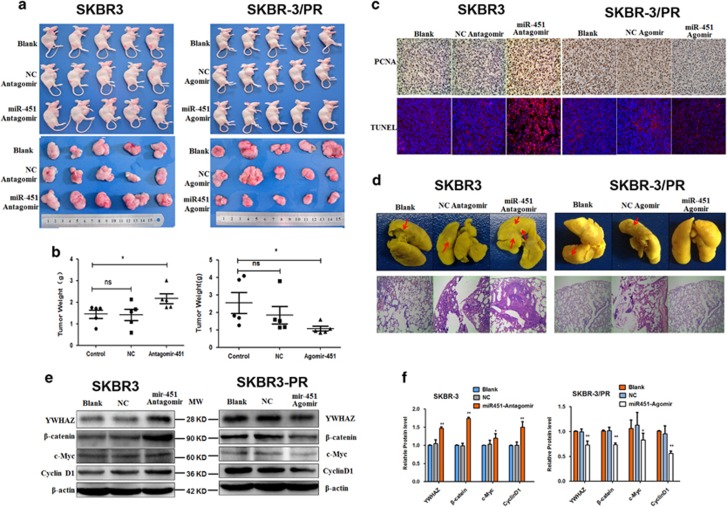
Effect of miR-451 on tumor growth in xenograft model and in drug-resistant xenograft model. (**a**) Representative images of tumor growth at 4 weeks after injection using miR-451 antagomir or miR-451-NC antagomir in SKBR3 xenograft model, miR-451 agomir or miR-451-NC agomir in SKBR3/PR drug-resistant xenograft model, respectively. SKBR-3 in the left panel and SKBR-3/PR in the right panel injected in the mice. (**b**) The mean tumor weights were measured at 4 weeks post injection. *P<0.05; *n*=5. Mean±S.D. (**c**) Proliferative activity assessed by anti-PCNA and monoclonal antibody in the tumors, original magnification × 200. (**d**) TUNEL assay was performed to observe the apoptotic cells. Original magnification × 200. (**e**) Representative photos of mouse lungs and images of the histological inspection of mouse lungs for the presence of microscopic lesions, original magnification × 400. (**f**) Western blotting analysis was performed to detect the expression of *β*-catenin, c-Myc and cyclinD1 *in vivo*, *β*-actin served as a loading control

In order to further validate the important role of miR-451 as a regulator on drug sensitivity in BC, we constructed xenograft model and drug-resistant xenograft by injecting SKBR3 cells and SKBR3/PR cells, respectively. As shown in Figure 10a (right panel), in the SKBR3/PR xenograft model, mice that received intratumoral injection of miR-451 agomir, the tumor size was significantly smaller than that of the negative control (NC) agomir group. Consistently, the mean weight of the tumors extracted from the miR-451 agomir group was lower than that of the NC agomir group (Figure 10b, right panel). The reverse effect was observed for the antagomir in SKBR3 xenograft model. The tumor size in the miR-451 antagomir group was much larger than those in the NC antagomir group (Figure 10a, left panel). The mean weight of the tumors extracted from the miR-451 antagomir group was significantly higher than those extracted from the NC antagomir group. Moreover, we also detected the PCNA expression level by immunohistochemically staining. Notably, we observed a decreased PCNA in the SKBR3/PR/miR-451 agomir-treated tumors compared with the blank group or the NC agomir group, while an increased PCNA in the SKBR3/antagomir-451 tumors compared with SKBR3/antagomir-NC group (Figure 10c). Apoptosis in tumor issues were detected by TUNEL assay. The results showed that the apoptotic rate of tumors developed from SKBR3/PR/miR-451 agomir groups was significantly higher than that of tumors developed from SKBR3/PR/agomir-NC following 4 weeks of treatment. The reverse effect was observed for the antagomir in SKBR3 xenograft model (Figure 10d). In addition, we found that the lung metastatic nodules were significantly decreased in the miR-451 agomir-treated mice compared with agomir-NC group, while increased lung metastatic nodules were significantly increased in the miR-451 antagomir-treated mice (Figure 10e). Finally, we explored the potential mechanism of the tumor-suppressive role of miR-451. In accordance with the results of the experiments performed *in vitro*, miR-451 decreased the mRNA and protein expression level of *β*-catenin and two WNT transcriptional targets, c-Myc and cyclin D1, were decreased concurrently (Figures 10f and g).

## Discussion

Chemotherapy has been widely used in treating many types of cancer, including BC. Although cancer treatments have achieved tremendous progress during the past decades, drug resistance remains a major unresolved obstacle in BC treatment. Recently, increasing evidences have shown that miRNAs play an important role in cancer chemotherapeutic resistance and can be potentially applied to reverse drug resistance. However, the mechanism of miRNA-mediated drug resistance is still poorly understood. In our current study, we explored the role of miR-451 in the development of drug resistance in BC.

Previous studies have indicated that miR-451 can act as a tumor suppressor in many various types of human cancers, including colorectal cancer, glioblastoma, hepatocellular carcinoma (HCC), and gastric cancer.^23, 24, 25^ For example, Bitarte *et al.* found that miR-451 caused a decrease in self-renewal, tumorigenicity, and chemoresistance to irinotecan of colon spheres in colorectal cancer.^23^ MiR-451 is identified to control the regulation of glioblastoma cell proliferation, invasion and apoptosis through the PI3K/AKT signaling pathway.^24^ Moreover, a recent study showed that miR-451 inhibited the migration of HepG2 and SK-Hep-1 cells via the regulation of ATF2 in HCC.^25^ In terms of the association of miR-451 and drug resistance, it has recently been reported that overexpression of miR-451 in gastric and colorectal cancer cells reduced cell proliferation and increased sensitivity to radiotherapy by regulating macrophage migration-inhibitory factor production.^26^ In addition, miR-451 has been reported to be correlated with chemosensitivity of BC cells to doxorubicin (DOX) via direct targeting of ABCB1 and NSCLC cells to cisplatin.^27, 28^ These studies revealed that miR-451 is not only useful as a marker, but also serves as a potential target for novel therapeutic strategies to overcome drug resistance and tumor growth. In current study, we found that miR-451 expression was downregulated in the paclitaxel-resistant human BC cell lines compared to their parental cell lines. Furthermore, miR-451 may be involved in cell proliferation, migration and apoptosis in cell lines. Our findings suggested that overexpression of miR-451 increased BC cell resistance to paclitaxel mainly through downregulating YWHAZ expression *in vitro* and *in vivo*. Importantly, identification of target genes regulated by miRNAs will be helpful to highlight the functions of miRNA in various life processes. Many studies have clearly indicated that one miRNA could control multiple genes, whereas one gene could also be regulated by multiple miRNAs.^29, 30, 31^ To determine the mechanism by which miR-451 regulates paclitaxel-resistant in BC, we predicted its target genes through bio-information analysis. YWHAZ codes for a known anti-apoptotic protein, 14-3-3*ζ*, is a member of the highly conserved a 14-3-3 zeta protein family.^32^ It has been shown to be overexpressed in >40% of advanced stage BCs, confer cancer cell apoptosis resistance, and correlate with poor survival in BC patients.^33^ YWHAZ serves as a pivotal factor that binds and stabilizes key proteins involved in signal transduction, cell proliferation, and apoptosis.^34^ Mao *et al.* reported that miR-544 is involved in cell-cycle regulation and suppresses cervical cancer cell proliferation, colony formation, migration and invasion in a manner associated with YWHAZ downregulation in cervical cancer.^35^ More importantly, YWHAZ is involved in drug resistance in BC.^36^ YWHAZ was a key signal molecule in doxorubicin resistance by reducing activation of the p38MAPK signal pathway in MCF-7/ADR. In line with these findings, our results showed that knockdown of YWHAZ promotes cell apoptosis, inhibits cell migration, and invasion in PR cells. We also noticed an inverse correlation between miR-451 and YWHAZ in BC *in vitro* and *in vivo*.

Previous study have demonstrated that combination of miRNA and siRNA-based treatment could afford improved dual inhibition of a target protein.^37^ George Calin and his colleagues reported that dual targeting of EphA2 using siRNA-EphA2 and miR-520d-3p exhibits synergistic inhibition of EphA2 and significantly augments tumor regression compared to either monotherapy alone both *in vitro* and *in vivo.*^38^ In this study, co-treatment with miR-451 mimics and YWHAZ-siRNA significantly enhanced YWHAZ knockdown in both SKBR3/PR and MCF-7/PR cells. However, the combination therapy showed synergistic inhibition of cell invasion (ratio of expected: observed Fa is 1.2 for SKBR3/PR and 1.4 for MCF-7/PR cells respectively), but not in migration. These findings provide a novel strategy for combining miRNA and siRNA therapy for the treatment of BC.

YWHAZ/*β*-catenin complex is involved in drug resistance in cancer metastasis. Chen *et al.* found that overexpression of YWHAZ promoted lung cancer cell proliferation, migration, and invasion *in vitro*, as well as tumorigenesis and metastasis *in vivo*. *β*-catenin is a central effector of Wnt signaling in tumorigenesis and metastasis.^21^ Another study have shown that upregulation of miR-200a downregulated the expression of *β*-catenin and affected the activity of the Wnt/*β*-catenin signaling pathway.^39^ In line with the role of miRNAs in regulating Wnt/*β*-catenin, we found that upregulation miR-451 could decrease *β*-catenin expression in PR cells and in xenograft mouse model. In particular, knockdown of YWHAZ by siRNA displayed a consentaneous phenomenon with the effect of miR-451 in PR cells.

**Figure 11:**
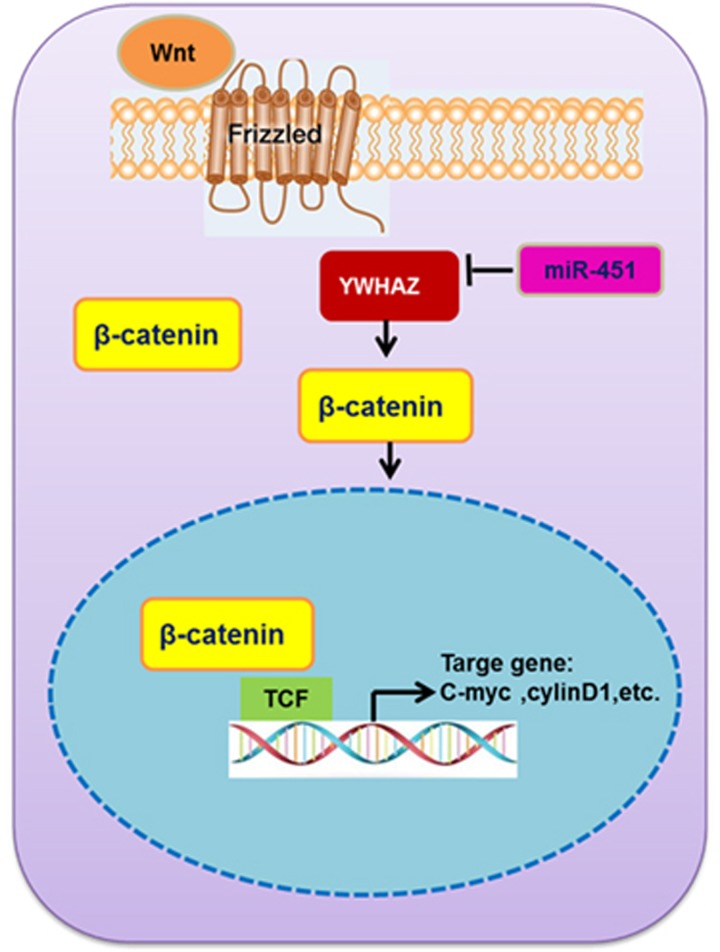
A schematic model depicting miR-451 regulates paclitaxel resistance in breast cancer

As *β*-catenin is a critical component of the well-studied Wnt/*β*-catenin signaling pathway, when Wnt/*β*-catenin signaling is activated, *β*-catenin degradation is inhibited, Wnt/*β*-catenin catenin increase in the cytoplasm and shift to the nucleus, then activate the target genes, including c-Myc, cyclin D1 and MMP-7, and so on, eventually lead to cell malignant transformation and tumorigenesis.^40, 41, 42^ C-Myc is a target gene of the Wnt signaling pathway. Under normal circumstances, c-Myc gene does not shown transcriptional activity or low levels of expression. Cyclin D1 is another target of Wnt signaling pathway. Fang found that miR-33a negatively regulated the downstream genes c-Myc and cyclin D1 of *β*-catenin in Wnt/*β*-catenin pathway, and also inhibited cells growth upon exposure to AFB1.^43^ However, wang reported that miR-181b inhibitors could increase the expression of c-Myc and cyclin D1, and reduce p27 expression in A549 cells.^44^ This study suggested that miR-451 could decrease the mRNA and protein expression level of *β*-catenin, c-Myc and cyclin D1. As shown in Figure 11, miR-451 negatively regulated the downstream genes c-Myc and cyclin D1 of *β*-catenin in Wnt/*β*-catenin pathway.

In summary, we demonstrated that miR-451 suppressed migration and invasion in BC *in vitro* and *in vivo*. Furthermore, miR-451 may induce apoptosis and cell-cycle arrest of PR cells through directly targeting the YWHAZ/*β*-catenin signaling pathway. MiR-451 and YWHAZ was negative correlated, and associated with chemosensitivity to paclitaxel and metastasis in animal model. Our findings provided further evidence that miR-451 might be considered as important and potential target for the diagnosis and treatment of paclitaxel-resistant BC.

## Materials and methods

### Cell culture

The human BC cell lines MCF-7, SKBR3 were cultured in Dulbecco’s modified Eagle’s medium (DMEM; Gibco, Gaithersburg, MD, USA) supplemented with 10% fetal bovine serum. Cells were incubated in atmosphere containing 5% CO_2_ at 37 °C. To establish PR cell lines, cells were continuously exposed to increased concentration for more than 6 months until cells displayed resistance to paclitaxel. MCF-7/PR and SKBR3/PR cells displayed resistance to cell growth inhibition of 10 *μ*g/ml paclitaxel and 25 *μ*g/ml paclitaxel, respectively.^45^ The resistant cells were maintained in culture medium with 10 *μ*g/ml or 25 *μ*g/ml paclitaxel.

### Cell transfection

The miR-451 mimic, and its NC, miR-451 inhibitors and its NC were purchased from Shanghai Gene Pharma Cells. siRNA specific for YWHAZ was chemically synthesized from Shanghai GenePharma Company. Cells were seeded in six-well plates and transfected with miR-451 mimics, miR-451 inhibitors or YWHAZ-siRNA using Lipofectamine 2000 (Invitrogen, Carlsbad, CA, USA) according to manufacturer’s protocol. After the indicated periods of incubation, the expression of miR-451/YWHAZ and other target genes was assessed with qRT-PCR detection system and western blot apparatus.

### Wound healing assay

The BC cells and BC PR cells were seeded in six-well plate until the cells reached to 90–95% confluency. The scratch wound was generated in the surface of the plates using a pipette tip in cells with miR-451 mimics, miR-451 inhibitors transfection or YWHAZ siRNA treatment. Photographic images were taken at 0 and 24 h. The images were then analyzed using Image J software.

### Transwell migration and invasion assays

The migration of cells and PR cells was conducted using a 24-well transwell chamber (Corning) with gelatin-coated polycarbonate membrane filter. The invasive capacity of cells was performed using transwell precoated with matrigel (BD Biosciences, San Jose, CA, USA). After incubation for 24 h, the upper surfaces of the transwell chambers were scraped with cotton swabs, and the migrated and invaded cells were fixed with 4% paraformaldehyde, and then stained with Giemsa solution. The stained cells were photographed and counted under a light microscope in five randomly selected fields.

### Apoptosis and cell-cycle analysis

Apoptosis were assayed using the multifunctional Muse Annexin V and Dead Cell kit (Millipore, Billerica, MA, USA) according to the user’s guide and the manufacturer’s instructions. Briefly, the BC cells and BC PR cells were harvested and washed with phosphate-buffered saline (PBS) twice. Then, 5 *μ*l of FITC-labeled enhanced annexin V and 5 *μ*l (20 *μ*g/ml) of propidium iodide were added to a 100 *μ*l cell suspension. After incubation in the dark for 15 min at room temperature, the samples were immediately analyzed by Muse Cell Analyzer (Merck, Millipore). All experiments were performed in triplicate. The cell-cycle distribution analysis was measured using a Muse Cell Cycle Assay Kit (Merck, Millipore) according to the manufacturer’s instructions. Cells were trypsinized, washed with PBS and fixed in 70% ethanol overnight. Then, cells were centrifuged, washed with PBS, dissolved in 200 *μ*l of Muse cell cycle assay kit, and analyzed by Muse Cell Analyzer (Merck, Millipore).

### RNA extraction, reverse transcription and real-time PCR quantification

Total RNA was extracted from cells using Trizol reagent (Invitrogen) according to the manufacturer’s protocols. One microgram of RNA was treated with 1 *μ*l DNase I (Fermentas, St. Leon-Rot, Germany) to remove DNA contamination. After determining RNA concentration and purity by using NanoDrop ND-8000 (Thermo Fisher Scientific, Waltham, MA, USA), the cDNA was synthesized using a reverse transcription kit (TaKaRa, Dalian, China) and quantitative real-time PCR was carried out using the SYBR premix Ex TaqII kit (TaKaRa) in an ABI 7500HT fast real-time PCR System (Applied Systems, Foster City, CA, USA) according to the manufacturer’s instructions, respectively. We used melting curves to monitor non-specific amplifications. Relative expression level was computed using 2^−ΔΔCt^ method. Each PCR amplification was performed in triplicate to verify the results. Gene expression levels were normalized to the expression of GAPDH, and miRNA expression levels were normalized to the expression of small nuclear U6 RNA The primers for the target genes in the study were the following: YWHAZ (forward: 5′-ACT TTT GGT ACA TTG TGG CTT CAA-3′ reverse: 5′-CCG CCA GGA CAA ACC AGTAT-3′); *β*-catenin (forward:5′-GGC TAC TGT TGG ATT GAT TCG AA-3′ reverse:5′-GCT GGG TAT CCT GAT GTGCAC-3′); c-Myc (forward: 5′-GCGACTCTGAGGAGGAACA-3′ reverse: 5′-TGAGGACCAGTGGGCTGT-3′); Cyclin D1 (forward: 5′-AGGAGAACAAACAGATCA-3′ reverse: 5′-TAGGACAGGAAGTTGTTG-3′); GAPDH (forward: 5′-CAG CCT CAA GAT CAT CAGCA-3′ reverse: 5′-TGT GGT CAT GAG TCC TTCCA-3′).

### Luciferase reporter assay

The miR-451 response element (wild type or mutated) in the 3′-UTR of YWHAZ was amplified by PCR and cloned into pMIR-REPORT (Ambion, Austin, TX, USA) with firefly luciferase. Cells treated with control, miR-451 mimics, or miR-451 inhibitors were cotransfected with wild-type or mutants of YWHAZ 3′-UTR luciferase reporters together with Renilla plasmid. Forty-eight  hours after transfection, the firefly and Renilla luciferases were assayed according to the manufacturer's instructions (Promega, Madison, WI, USA), and the firefly luciferase activity was normalized to that of Renilla luciferase. Each experiment was repeated in triplicate.

### *In vivo* xenograft model studies

BALB/c female nude mice of 4 weeks of age were purchased from SLAC Laboratory Animals Co. (Shanghai, China). Experiments involving animals were approved by the Animal Care and Use Committee of Bengbu Medical College. All the nude mice were equally divided into six groups (5 mice/group). SKBR3 and SKBR3/PR cancer cells were resuspended in 1 : 1 PBS and matrigel mixture and injected into the fat pads of second mammary glands of mice as follows: 4 × 10^6^ SKBR3 cells/100 *μ*l per point for each nude mice (15 total), 4 × 10^6^ SKBR3/PR cells/100 *μ*l per point for each nude mice (15 total). From the 10th day after cell injection, we confirmed the engraftment of the tumors. SKBR3 xenograft-bearing mouse model were randomized into three groups (*n*=5), five mice were injected with 5 nM micr^OFFTM^ antagomir-451; five mice were injected with 5 nM antagomiR NC, and the remaining five mice received PBS as a control. Also, at the same time, for SKBR3/PR xenograft-bearing mouse model, five mice were injected with 5 nM micr^ONTM^ agomir-451, five mice were injected with agomiR NC and the remaining five mice received 100 *μ*l PBS as a control. The mice were humanely sacrificed on day 38, and the tumors were removed, weighed, and photographed.

The xenograft tumors were excised neatly, weighed and subsequently processed for H&E staining, and preparation of protein extracts for western blotting. Tumor proliferation was assessed by PCNA stain and tumor apoptosis were assayed using TUNEL staining as described previously.^46^ The lung tissues were immersed and fixed in bouin compound for 24 h and destained with absolute ethanol for detecting the metastatic nodules in the lung surface of mice.

### Western blot analysis

Cells and PR cells transfected with miR-451 mimics, miR-451 mimics NC, miR-451 inhibitor, miR-451 inhibitor NC or YWHAZ-siRNA were rinsed with PBS (pH 7.4) and then harvested and lysed in RIPA lysis buffer (Beyotime, Shanghai, China) with freshly added PMSF (Beyotime) for 30 min on ice. Tissue samples were frozen solid with liquid nitrogen, ground into a powder and lysed in RIPA lysis buffer supplemented with PMSF on ice for 30 min. Sonication was used to facilitate cell lysis. The protein concentration was quantified using Bio-Rad protein assay kit (Bio-Rad Laboratories, CA, USA). Primary antibodies against YWHAZ, *β*-catenin, c-Myc and Cyclin D1 and *β*-actin were bought from Santa Cruz Biotechnology (Santa Cruz, CA, USA). Quantification of protein bands was performed using the Image J software.

### TCGA data analysis

The results of integrated analysis data of miRNA expression data for 104 corresponding BC patients (tumor and/or adjacent normal tissue) were downloaded from the TCGA Data portal. The expression analyses were carried out using BRB-ArrayTools (version 4.5, National Cancer Institute, Bethesda, MD, USA).

### MicroRNA array and data analysis

Microarray was performed at KangChen Bio-tech (Shanghai, China). Total RNA was extracted from MCF-7 and MCF-7/PR cells using TRIzol (Invitrogen) and miRNeasy mini kit (QIAGEN, Denmark) according to manufacturer’s instructions. The RNA quality and quantity were measured by NanoDrop ND-8000 (Thermo Fisher Scientific). The RNA sample was labeled with Cy3 or Cy5 fluorescent dyes by miRCURY Array Power Labeling kit (Exiqon, Vedbaek, Denmark) according to the manufacturer's protocols. Gene Pix 4000B scanner and GenePix Pro 6.0 software (AxonInstruments, Union City, CA, USA) were used to acquire images. Background subtraction and normalization were performed. Two-fold or larger change was set as a threshold of significant difference.

### Statistical analysis

All experiments were repeated at least three times. Data are presented as mean±standard deviation (S.D.) and analyzed for significance using GraphPad Prism 6 software (La Jolla, CA, USA) Two-tailed *t*-test was used when two groups were compared. *P<0.05, **P<0.01, ***P<0.001 were considered to be statistically significant.

## Publisher’s Note:

Springer Nature remains neutral with regard to jurisdictional claims in published maps and institutional affiliations.
