# Supplementary figures and images for: Involvement of miR-451 in resistance to paclitaxel by regulating YWHAZ in breast cancer
Source: Cell Death Dis. 2017 Oct 5;8(10):e3071–. doi: 10.1038/cddis.2017.460 (PMC5680582; doi:10.1038/cddis.2017.460)

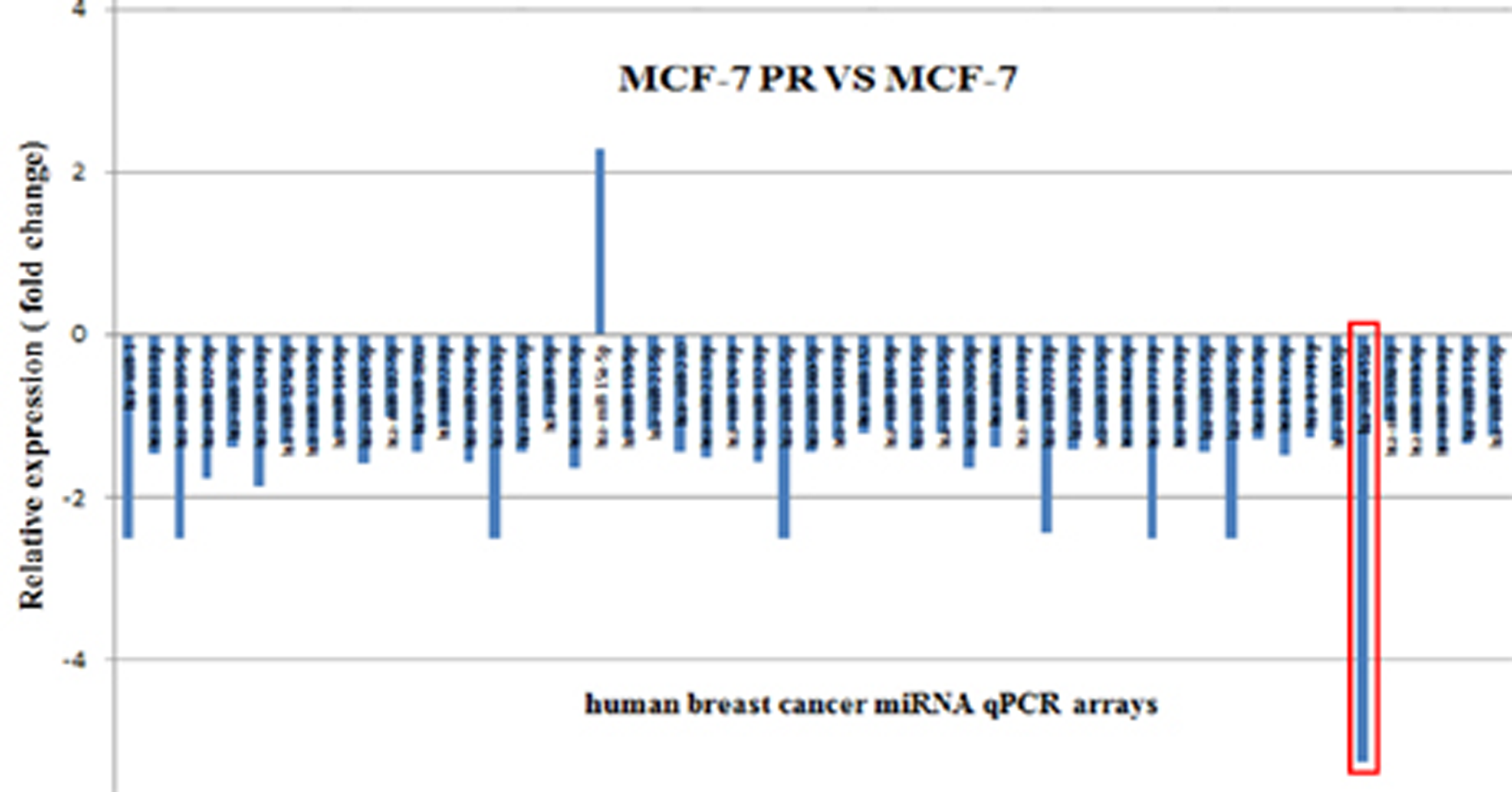

Supplement: Supplementary Figure S1 [file cddis2017460x1.tif]
