# Supplementary material for: Involvement of miR-451 in resistance to paclitaxel by regulating YWHAZ in breast cancer
Source: Cell Death Dis. 2017 Oct 5;8(10):e3071–. doi: 10.1038/cddis.2017.460 (PMC5680582; doi:10.1038/cddis.2017.460)
Supplement: Supplementary Figure Legend [file cddis2017460x2.docx]

**Figure S1** MiRNAs differentially expressed in MCF-7 and the MCF-7/PR cells by using miRNA microarray.
